# Spatial Guidance Overrides Dynamic Saliency in VR: An Eye-Tracking Study on Gestalt Grouping Mechanisms and Visual Attention Patterns

**DOI:** 10.3390/jemr18050037

**Published:** 2025-08-25

**Authors:** Qiaoling Zou, Wanyu Zheng, Xinyan Jiang, Dongning Li

**Affiliations:** 1 School of Design, Jiangnan University, Wuxi 214122, China; 7220306012@stu.jiangnan.edu.cn; 2 School of Digital Technology & Innovation Design, Jiangnan University, Wuxi 214401, China; 6220306059@stu.jiangnan.edu.cn; 3 School of Theatre and Film, Shanxi Normal University, Taiyuan 030032, China; jiangxinyan@sxnu.edu.cn

**Keywords:** virtual reality, spatial guidance, dynamic saliency, Gestalt, eye-tracking

## Abstract

(1) Background: Virtual Reality (VR) films challenge traditional visual cognition by offering novel perceptual experiences. This study investigates the applicability of Gestalt grouping principles in dynamic VR scenes, the influence of VR environments on grouping efficiency, and the relationship between viewer experience and grouping effects. (2) Methods: Eye-tracking experiments were conducted with 42 participants using the HTC Vive Pro Eye and Tobii Pro Lab. Participants watched a non-narrative VR film with fixed camera positions to eliminate narrative and auditory confounds. Eye-tracking metrics were analyzed using SPSS version 29.0.1, and data were visualized through heat maps and gaze trajectory plots. (3) Results: Viewers tended to focus on spatial nodes and continuous structures. Initial fixations were anchored near the body but shifted rapidly thereafter. Heat maps revealed a consistent concentration of fixations on the dock area. (4) Conclusions: VR reshapes visual organization, where proximity, continuity, and closure outweigh traditional saliency. Dynamic elements draw attention only when linked to user goals. Designers should prioritize spatial logic, using functional nodes as cognitive anchors and continuous paths as embodied guides. Future work should test these mechanisms in narrative VR and explore neural correlates via fNIRS or EEG.

## 1. Introduction

Virtual Reality (VR) is reshaping artistic expression by offering immersive, 360-degree experiences that redefine narrative perception [1,2]. Unlike traditional two-dimensional films, where framing and montage guide viewer focus, VR transforms this process by requiring viewers to self-direct attention in complex visual environments [3,4]. This shift raises key questions about how audiences organize visual information without directorial cues, challenging the visual cognitive system and prompting a reevaluation of Gestalt psychology in new media contexts [5]. Gestalt theory, centered on principles like proximity, similarity, continuity, closure, and common fate [6,7], offers a foundational framework for understanding VR visual narratives. Although well-established in traditional art [8,9], its application in VR still requires thorough investigation.

VR’s 360-degree immersive narrative can shape how viewers integrate visual information during the experience. Neuroaesthetic research highlights the complexity of this process, showing that artistic perception is closely tied to the brain’s visual processing mechanisms [10,11]. When engaging with art, the visual system automatically performs multi-level integration, from basic feature detection to advanced semantic interpretation [12]. The immersive nature of VR amplifies this integration by activating a wider array of neural circuits than traditional media, potentially enhancing both cognitive and emotional engagement with the artwork [13]. However, despite theoretical support from cognitive neuroscience and perceptual psychology, there remains a notable lack of empirical research specifically focused on how VR environments influence the foundational grouping processes described in Gestalt psychology, processes that are essential to perceptual organization and meaning-making within visual experiences; this gap highlights the need for systematic investigation into how established perceptual theories adapt and operate within the dynamic, interactive contexts of VR art.

On a practical level, VR film production faces unprecedented challenges in guiding audience attention within immersive environments, where traditional cinematic techniques lose their efficacy [14]; in response, avant-garde directors have begun experimenting with innovative strategies, such as manipulating lighting and shadow [15], incorporating spatial audio [16], and implementing dynamic guidance mechanisms [17], to replicate or replace the role of conventional visual cues, though the effectiveness of these alternatives remains in urgent need of empirical validation. As panoramic immersion disrupts linear storytelling control, VR creators are increasingly turning to Gestalt principles for structured, professional solutions, such as applying the law of continuity to craft intuitive gaze pathways or utilizing the law of common fate to establish dynamic focal points that guide visual attention organically [18]. However, artistic experiences are shaped by individual differences, with artistic training affecting visual processing, an effect potentially amplified in VR [19]. From an evolutionary perspective, humans’ preferences for certain visual patterns, such as flowing water, open landscapes, and symmetry, may have adaptive biological roots [20,21]. VR films, with their immersive and dynamic environments, can easily trigger these ingrained perceptual tendencies [22]. Additionally, Gestalt principles are influenced by both individual and cultural factors, which shape the strength and nature of perceptual grouping. Thus, creating perceptually engaging VR films requires considering biological predispositions, personal artistic backgrounds, and cultural visual frameworks.

Therefore, building on the recent integration of eye-tracking technology in VR research [23,24] this study seeks to investigate the applicability of Gestalt grouping principles within dynamic scenes of VR films by leveraging eye-tracking metrics to analyze how immersive environments influence visual organization; specifically, the study aims to uncover the underlying mechanisms through which VR alters grouping efficiency and to examine the correlation between audience members’ subjective experiences and the manifestation of grouping effects, thereby contributing empirical insights into perceptual organization in immersive cinematic contexts.

Based on this, the specific objectives of this study are as follows:

**O1:**  *To assess the applicability and relative strength of key Gestalt grouping principles, Proximity, Similarity, Continuity, Closure, and Common Fate, within dynamic VR film scenes by analyzing eye-tracking patterns that reflect how viewers visually attend to and organize complex immersive stimuli*.

**O2:**  *To examine how specific dynamic characteristics inherent to VR scenes influence the efficiency of perceptual grouping, as quantified through eye-tracking metrics that capture viewer attention, fixation behavior, and visual processing patterns within immersive environments*.

**O3:**  *To explore the relationship between viewers’ subjective reports of scene coherence, engagement, and presence and the objectively measured efficiency of Gestalt grouping effects, as derived from eye-tracking data, in order to understand how perceptual organization aligns with individual experiential responses in immersive VR settings*.

## 2. Literature Review

### 2.1. Research Progress on Visual Perception of VR Films

Lamb et al. [25] demonstrated that, compared to traditional flat films, viewers in VR environments exhibit significantly heightened spatial selectivity in their visual exploration behavior, indicating that within immersive 360-degree panoramic scenes, attention becomes more proactive and self-directed, with viewers focusing on personally selected spatial zones rather than following the director’s pre-established framing through techniques like shot composition and editing, as seen in conventional filmmaking [26]; subsequent studies have quantified this tendency [27], with Sitzmann et al. [28] reporting that viewers’ gaze tends to cluster within limited sectors of the VR scene, and Lee et al. [29] confirming that specific spatial cues, such as lighting variation and sound localization, can effectively steer attention. In terms of gaze distribution, research has illuminated the unique attentional dynamics of VR viewing; for instance, Holm et al. [30] found that dynamic visual elements, including moving objects, character actions, and scene transitions, play a more potent role in redirecting viewer focus [31], a finding supported by experimental studies showing that dynamic cues like animated objects and moving light spots are particularly effective at guiding users toward key narrative or interactive elements in complex immersive environments [32,33]. Significant progress has been made in exploring Gestalt grouping principles in VR environments. Welchman et al. [34] were the first to systematically examine how depth information affects perceptual grouping, showing that organization mechanisms in 3D space differ fundamentally from those in 2D. This finding has opened new research directions, prompting a reassessment of Gestalt principles in immersive media. However, current studies face limitations in stimulus selection, as most rely on short experimental clips [35], with few focusing on VR films featuring coherent narratives.

### 2.2. VR Adaptive Correction of Gestalt Principles

Gestalt psychology, a foundational theory that explains the organization of human visual perception, has been extensively validated within traditional two-dimensional media contexts [36]; however, the emergence of VR has introduced profound challenges to these principles, prompting researchers to systematically reconsider and revise classical Gestalt concepts in light of immersive spatial dynamics [37]. Initial empirical findings questioned the applicability of the proximity principle in VR, revealing that grouping effects become significantly stronger when elements differ in actual depth rather than merely in their two-dimensional projection [38,39]. Similarly, the principle of similarity acquires greater complexity in VR environments; for instance, research by Morimoto et al. [40] demonstrated that color-based similarity effects are often weakened by depth cues, while shape-based similarity remains relatively robust, particularly when viewers are tasked with interpreting abstract symbols [41]. Among all the principles, closure undergoes the most striking adaptation in VR. Stein et al. [42], using eye-tracking data, showed that users in VR environments actively utilize head movements to acquire supplementary visual input, thereby mentally completing fragmented three-dimensional structures in ways not observed in flat media. Furthermore, the inherently dynamic nature of VR introduces a temporal dimension into the perceptual grouping process, with groundbreaking research [43] revealing that Gestalt reorganization in VR is subject to a notable neural delay, significantly longer than in two-dimensional settings, likely due to the increased cognitive load involved in processing three-dimensional spatial information [44,45], a finding that underscores the need to reinterpret traditional visual theories within the immersive frameworks of modern media.

### 2.3. Empirical Evidence of Eye-Tracking of Gestalt Principles

Eye-tracking technology presents an ideal methodological approach for investigating the applicability of Gestalt principles within VR environments, as it enables precise documentation of visual attention through metrics such as fixation points, saccades, and pupillary responses during immersive viewing experiences [46,47]; however, the distinct characteristics of VR necessitate the consideration of new technological challenges and analytical frameworks, particularly as traditional two-dimensional assumptions may not fully apply. A critical factor in VR research is the interplay between eye movements and head movements, which can reveal complex interactions between bottom-up sensory input and top-down cognitive control in visual perception [48]. Unlike conventional 2D settings, VR introduces the need to account for three-dimensional spatial perception when analyzing Gestalt grouping, with evidence showing that depth information can enhance the “common fate” principle, where elements moving together are perceived as a group, while simultaneously weakening the effectiveness of the “similarity” principle due to spatial disparities [49,50]. These phenomena can be quantified using fixation clustering analyses, comparing gaze distributions across identical scenes presented in both 2D and VR formats to assess the modulation of grouping effects by immersive depth [51]. Moreover, VR’s interactive nature compels researchers to reevaluate the operational definition of “fixation,” traditionally defined as a gaze held for over 100–200 ms [52,53]; in immersive environments, the user’s freedom of head movement often blurs the boundary between fixations and saccades, complicating interpretation [54]. To address this, recent studies advocate for the integration of head-tracking data alongside eye-tracking metrics, enabling a more nuanced distinction between active visual exploration and passive gaze behavior in dynamic VR settings [55] and thus offering a more comprehensive understanding of perceptual organization under immersive conditions.

### 2.4. Research Hypotheses

In summary, the existing body of literature suggests that VR environments significantly transform viewers’ visual exploration behavior by fostering heightened spatial selectivity and increased sensitivity to dynamic cues, indicating that perceptual organization in immersive settings is heavily influenced by active processing guided by spatial cognitive intentions; this shift challenges the classical Gestalt grouping principles, which require adaptive reinterpretation to remain applicable in three-dimensional, interactive contexts. Despite theoretical advancements, there is a notable lack of systematic empirical studies examining how spatially and dynamically distinct regions within VR films compete for and direct visual attention under these revised principles, as well as how subjective experiences, such as coherence, presence, and engagement, are reflected in eye movement patterns. Crucially, the fundamental question of whether spatial intention and structural guidance take precedence over classical dynamic salience in shaping attention allocation remains unresolved and demands urgent empirical verification to advance both perceptual theory and immersive media design.

Therefore, to directly address the identified research gaps, namely, the competition between spatial and dynamic features for attention under revised Gestalt principles, the reflection of subjective experience in eye movement patterns, and the critical question of whether spatial guidance precedes dynamic saliency, this study formulates five hypotheses that systematically target core aspects emerging from prior theoretical and empirical challenges: (1) the dominance of significant spatial nodes (H1) and continuous structures (H2) over isolated dynamic elements in governing the flow of visual attention; (2) the anchoring function and temporal characteristics of the proximity principle (H3) during the initial phase of VR scene exploration; (3) the reduced effectiveness of similarity-based grouping (H4) within the spatially fragmented and depth-rich 360° VR environment; (4) the conditional nature of dynamic grouping based on the principle of common fate (H5), which is hypothesized to require cognitive alignment with user goals in order to be effective; and (5) implicitly, the correlation between successful spatial grouping, outlined in H1 and H2, and viewers’ subjective experiences of narrative coherence and immersive engagement. Collectively, these hypotheses aim to empirically test the proposition that spatial-intentional guidance serves as a dominant organizing force in VR film perception, superseding the classical influence of dynamic visual salience.

Drawing on the adaptive applications of Gestalt psychology in VR and recent empirical developments in VR visual perception, this study formulates the following hypotheses to address its three core objectives:

**H1:**  *In VR films, spatial nodes that serve as potential targets or endpoints attract increased visual attention and undergo more in-depth cognitive processing*.

**H2:**  *In VR films, continuous spatial structures more effectively guide viewers’ visual scanning paths and organize the flow of attention than isolated dynamic elements*.

**H3:**  *This study also investigates whether spatial areas positioned closer to the viewer’s body are more likely to attract visual attention in VR environments*.

**H4:**  *In VR films, elements that share similar spatial structures are more likely to induce perceptual grouping effects than isolated dynamic components, thereby enhancing the capture and retention of audience attention*.

**H5:**  *In VR films, the coherent movement of dynamic elements automatically activates the perceptual grouping principle known as the law of common fate, which effectively captures the audience’s attention*.

The relationship between objective, hypotheses, literature, and purpose is shown in Table 1 below:

## 3. Materials and Methods

### 3.1. Experimental Design

**Figure 1:**
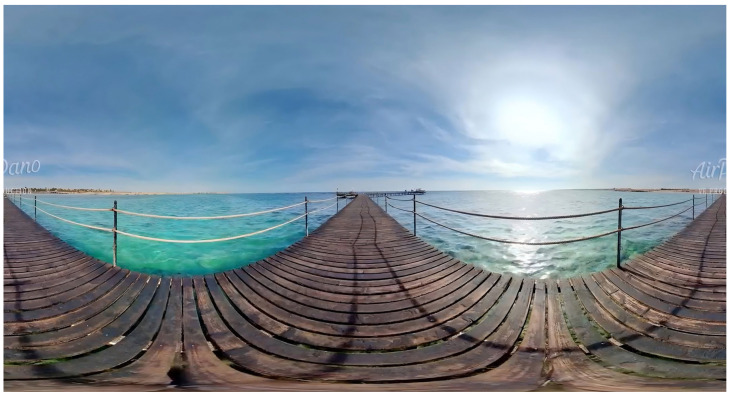
Screenshot of the controlled-design, non-narrative VR experimental film used as the visual stimulus in the eye-tracking study.

This study utilized eye-tracking technology to test the proposed hypotheses. A 29-s VR experimental film was selected for this purpose (Figure 1). The film features key visual elements, including docks, bridges, islands, bodies of water, and people. It is a non-narrative film shot using fixed camera positions and contains no editing effects. This controlled design was intended to eliminate confounding variables, such as narrative structure and auditory cues, that might otherwise influence participants’ visual attention during the experiment [56]. To contextualize our findings on VR-specific visual processing and to reinforce claims about the distinctive affordances of immersive environments, it is essential to consider existing research comparing visual attention in traditional 2D displays and VR. Although the present study focused solely on the VR condition and did not include a 2D control group viewing the same stimulus, prior empirical studies offer a solid foundation for interpreting our results. Extensive research on 2D media has consistently demonstrated the effectiveness of dynamic saliency cues and Gestalt principles, such as similarity and the law of common fate, in capturing and directing attention under controlled conditions [57,58,59]. However, comparative studies indicate that the immersive characteristics of VR, such as stereoscopic depth perception, a 360° field of view, head-tracked interactivity, and an intensified sense of spatial presence, profoundly reshape attentional dynamics [60].

### 3.2. Participants

The research team randomly recruited 42 volunteers, aged between 18 and 36 years, to participate in the study. The sample consisted of 25 males and 17 females. An a priori power analysis was conducted using G*Power 3.1 to determine the minimum sample size required to detect medium effects (f = 0.25) in a one-way ANOVA involving four conditions (AOI1–AOI4). With a significance level of α = 0.05 and a desired statistical power of 0.80, the analysis indicated that a minimum of 36 participants would be necessary. Our final sample of 42 participants exceeded this threshold, ensuring sufficient statistical power to detect meaningful effects in the eye-tracking metrics. Participant engagement and comprehension were further verified through direct observation and verbal questioning. All participants were currently enrolled students at Jiangnan University. They possessed normal cognitive and motor functions, with no history of mental illness or substance abuse, and were not majoring in film or related disciplines. Participants were required to have normal or corrected-to-normal vision and be free from eye conditions such as color blindness or color deficiency. It was learned through observation and verbal questioning that all participants signed informed consent forms to safeguard their rights. All data collection methods ensured the privacy and security of personal information, without disclosing any identifiable details of the participants. If identifiable images of participants are to be included in publications, additional written consent specific to image usage will be obtained retrospectively from those individuals prior to publication.

### 3.3. Experimental Procedure

The experimental procedure was conducted individually in a closed and well-conditioned laboratory setting. Prior to the commencement of the experiment, each participant was mandated to sign a consent form and complete a questionnaire encompassing their gender, age, and basic VR skills, thereby obtaining their demographic characteristics (Table 2). Results showed that most participants had prior experience with VR movies and could interact independently. To ensure comprehension, participants without VR experience underwent training with five types of VR films before the experiment. If they failed to demonstrate normal cognitive understanding afterward, they were excluded. This approach minimized novice effects and filtered out unsuitable participants, helping to ensure data accuracy.

Observers briefly spoke with participants to ensure they were in a stable emotional and psychological state, free from symptoms like depression, irritability, or excessive excitement. Participants were then familiarized with the venue and assisted in wearing the VR headsets. After eye tracker calibration, the experiment began following these steps:

(1) Informing the subjects about the experimental procedure, with instructions to terminate the experiment immediately if they experienced dizziness or discomfort. During this step, participants were also informed of the experimental purpose and guided to pay particular attention to the narrative aspect of the VR movie.
(2) Participants underwent visual calibration to ensure the precision of the eye-tracking data.
(3) Participants commenced viewing the movie, with data collection taking place concurrently.

This study complies with the Declaration of Helsinki and was approved by the Institutional Review Board of Jiangnan University. Participants will receive gifts after the experiment as a token of appreciation. Two coordinators oversaw the entire process, ensuring smooth execution and assisting with VR equipment setup and other needs.

### 3.4. Experimental Equipment

This study used Tobii Pro Lab and HTC Vive Pro Eye VR to collect and analyze eye movement data. Tobii Pro Lab supports complex experimental designs and real-time, high-precision data collection and is fully compatible with VR stimuli. The HTC Vive Pro Eye, equipped with Tobii’s eye-tracking, features 3.5-inch dual AMOLED screens (2880 × 1600 resolution), 120 Hz output, 0.5–1.1° accuracy, and a 110° tracking field. Before the experiment began, each participant underwent eye tracking calibration using the nine-point calibration program built into the HTC Vive Pro Eye to ensure that the tracking accuracy was within the manufacturer’s claimed error range of 0.5–1.1°. All experimental procedures, data processing, and analyses were conducted using a Seven Rainbow X17 Pro Max laptop (Colorful Technology Co., Ltd., Shenzhen, China), equipped with an Intel Core i9-13900HX (Intel Corporation, Santa Clara, CA, USA) processor and an NVIDIA GeForce RTX 4080 (NVIDIA Corporation, Santa Clara, CA, USA) graphics card, ensuring high-performance computing and efficient handling of experimental data.

**Figure 2:**
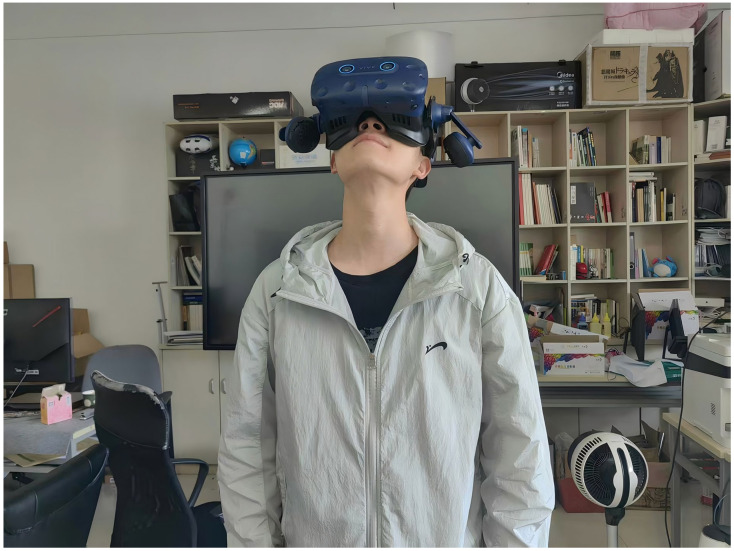
Experimental setup: A participant stands wearing the HTC Vive Pro Eye (Tobii-integrated, calibrated) within the tracking volume defined by the diagonally positioned base station, while the experimenter conducts real-time monitoring.

The experimenter was positioned at the center of the space where the base station was installed and could either stand or sit on a 360-degree rotating chair (Figure 2). The base station functions as a device that defines the boundaries of the virtual environment within the real-world setting. For this experiment, it was installed on the diagonal end face of the main setup to facilitate efficient operation and coverage. The experiment administrator remained in close proximity to both the participants and the equipment to ensure the smooth progression of the experiment, using a PC host to monitor the experimental status in real time.

### 3.5. Data Collection and Pre-Processing

**Figure 3:**
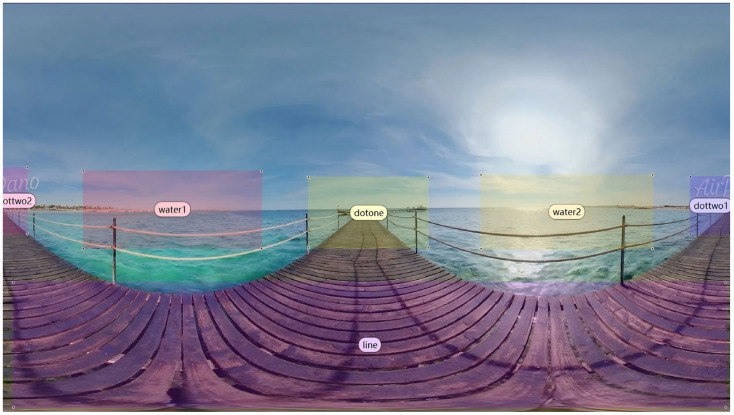
Dynamic AOI partitioning synchronized with temporal-spatial progression in the non-narrative 360° VR film.

Future research directions may also be considered in light of the findings. Various eye-tracking indicators offer insights into different dimensions of visual attention and cognitive load. Among these, fixation [61], saccades [62], and pupil diameter [63] are the most commonly used metrics for evaluating attention and cognitive processing. Before extracting these indicators, Areas of Interest (AOIs) within the VR film were defined. Given that the narrative unfolds within a 360° immersive environment, a dynamic AOI system was implemented (Figure 3), evolving in sync with the progression of the narrative. The AOIs were divided into four graphical zones: dock (AOI1—dotone), island (AOI2—dottwo1 and dottwo2), bridge (AOI3—line), and water (AOI4—water1 and water2).

Eye-tracking data indicators provide valuable insights into how audiences engage with film content. The Total Duration of Fixations (TFD) reflects the level of attention viewers allocate to a film, while Fixation Count (FC) and Saccade Action Count (SAC) offer dynamic information about shifts in visual attention [64]. Additionally, Time to First Fixation (TTFF) indicates how quickly specific elements capture initial attention, and Average Duration of Visit (ADV) reveals the depth of cognitive processing during focused viewing [65]. Eye-tracking heat maps and gaze trajectories further visualize the spatial distribution of viewers’ attention and the pathways of visual scanning during VR movie experiences. These visualization tools help identify visual hotspots and reveal which scenes or elements most effectively capture the audience’s attention [66,67]. They also illustrate how viewer attention may be diverted by certain segments and whether those segments contribute to frequent visual distractions [68]. Based on these considerations, we extracted the following metrics for each Area of Interest (AOI): TFD, FC, SAC, TTFF, and ADV. This was preceded by a preliminary review of the raw eye-tracking data, and we eliminated two sets of invalid data due to their chaotic and illogical eye-tracking information, ultimately retaining 40 valid datasets. Data validity was assessed through a combination of quantitative metrics and qualitative inspection. Key quantitative indicators included sample loss rate, tracking ratio, and spatial accuracy (mean error) obtained from calibration validation. Datasets were excluded if they exhibited a sample loss greater than 20%, a tracking ratio below 70%, or a calibration mean error exceeding 1.0° of visual angle. In addition to these criteria, datasets showing fundamentally illogical patterns, such as erratic gaze behavior unrelated to the stimuli, physiologically implausible saccades, or prolonged gaze directed outside the display area, were considered invalid and subsequently excluded from analysis. Data analysis was conducted using SPSS version 29.0.1, incorporating descriptive statistics, analysis of variance (ANOVA), and correlation analysis. In addition, by integrating the generated eye-tracking heat maps and gaze trajectories, we examined the influence of the four graphical regions (AOIs) on audience visual attention.

### 3.6. Operationalization of Objectives and Hypotheses

Based on the correspondence between the study hypotheses and the eye movement metrics, the experimental design operationalized the hypothesis validation as follows:

To test H1, the depth of cognitive processing at spatial endpoints was quantified using Total Duration of Fixations (TFD) and Fixation Count (FC) within the AOI1 region. For H2, the guiding effectiveness of continuous spatial structures was assessed using Saccade Action Count (SAC) and the linearity of eye movement trajectories, indicated by the dense distribution of gaze paths along the medial axis of the bridge. In evaluating H4, we analyzed differences in TFD, FC, and the clustering density of gaze points between the AOI2 and AOI4 regions to assess the attentional equivalence of structurally similar elements. To test H5, the attentional capture effect of dynamic elements was measured using SAC values and the spatial distribution of gaze points within AOI4, focusing on the degree of dispersion in eye movement trajectories. For H3, Time to First Fixation (TTFF) was employed to evaluate the speed at which attention was initially drawn to near-field regions. Additionally, the correlation between subjective experience and the grouping effect was examined through the Average Duration of Visit (ADV) as a physiological indicator of cognitive engagement, supported by correlation analyses between core eye-tracking metrics (TFD, FC, SAC) and time-synchronized subjective report data streams.

## 4. Results

### 4.1. Applicability of Gestalt Principles

**Figure 4:**
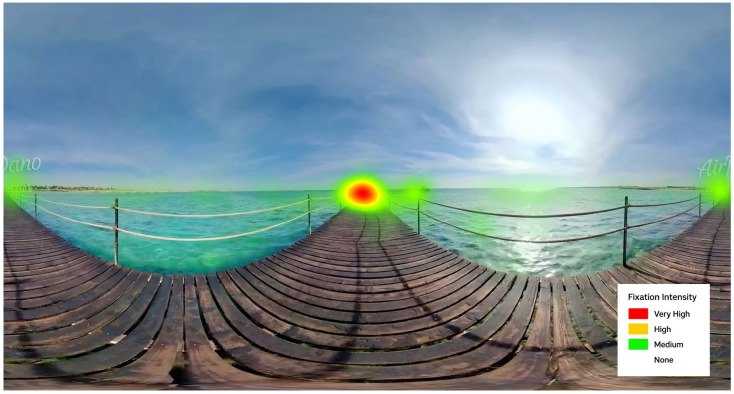
Eye-tracking heat map showing gaze intensity: red (very high), yellow (high), green (medium), transparent areas indicate no gaze points.

As a key terminal spatial node, AOI1 attracted significantly longer Total Fixation Duration (TFD). As shown in Table 3, its gaze duration exceeded that of AOI2 by 5085.200 ms and that of AOI4 by 5176.525 ms. In terms of Fixation Count (FC), AOI1 recorded a value 16.675 times higher than AOI4. The corresponding heatmap (Figure 4) further substantiates this dominance: AOI1 displays distinct red-yellow hotspots, indicating that it served as the primary visual focal point in the scene. These results suggest that viewers consistently identified AOI1 as the narrative endpoint. Collectively, these findings support Hypothesis H1, demonstrating that spatial nodes with terminal or closed meaning can surpass dynamic salience in effectively guiding visual attention.

**Figure 5:**
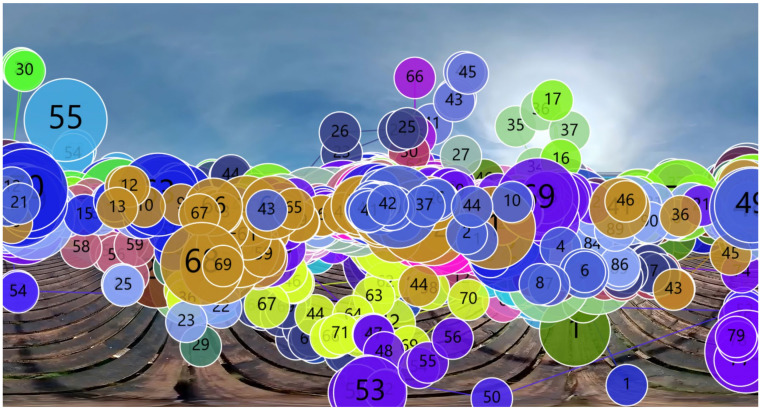
Eye-tracking trajectory map showing gaze parameters: trajectories represent scan paths, numbered points indicate fixation locations (numbered by temporal sequence, 1 = first).

The geometrically similar regions AOI2 and AOI4 did not exhibit significant differences across attention-related metrics. The difference in Total Fixation Duration (TFD) between the two was minimal (Δ = 91.325 ms, *p* = 0.998), and Fixation Count (FC) was also largely comparable (Δ = −1.625, *p* = 0.921). These results suggest that spatial dispersion within the 360° VR environment impeded perceptual grouping based on similarity. Eye-tracking plots (Figure 5) visually reinforce this conclusion, showing that gaze distributions across AOI2 and AOI4 were sparse and irregular, lacking the cohesive clustering typically associated with grouped attention. Collectively, these findings refute Hypothesis H4, indicating that in the absence of spatial association, similarity alone is insufficient to elicit effective Gestalt-based grouping effects in immersive VR contexts.

Although AOI4 exhibited consistent dynamic movement, it generated significantly fewer Saccade Action Counts (SAC) compared to the other regions. As shown in Table 3, the mean SAC difference between AOI1 and AOI4 was 9.850 units, highlighting notably reduced sweeping activity in the dynamic water region. Additionally, AOI4 evoked 3.35 fewer sweeps than the continuous structural region AOI3. Eye movement trajectories (Figure 5) further illustrate this effect: gaze paths within AOI4 were disorganized, with only a small number of gaze points containing sweeps directed toward the region. Most saccadic paths bypassed it entirely. These findings directly contradict the attentional capture predicted by the Gestalt Law of Common Fate, thereby refuting Hypothesis H5. The results suggest that motion alone is insufficient to attract attention in immersive environments; dynamic elements must be cognitively relevant to the viewer’s goals to effectively draw and sustain visual attention.

### 4.2. Examine VR’s Impact on Grouping Efficiency

As a tangible manifestation of the Gestalt principle of continuity, AOI3 elicited significantly more visual exploration activity compared to isolated dynamic elements. According to Table 4, the number of Saccade Action Counts (SAC) for AOI3 was substantially higher than that for AOI4, exceeding it by 3.350 units, representing a 3.3-fold increase. This marked difference in saccadic activity strongly suggests that the continuous linear structure of the bridge effectively organizes and guides the audience’s visual attention. Eye-tracking trajectory diagrams (Figure 5) further corroborate this finding: gaze paths densely align along the central axis of the bridge, forming a linear flow from the proximal starting point near the viewer’s body to the distal endpoint at the AOI1 bridge pier. This visual pattern confirms that continuous spatial structures are significantly more effective than isolated dynamic elements in directing attention flow, thereby providing empirical support for Hypothesis H2.

The first fixation time (TTFF) metric (as shown in Table 4) indicates that AOI1 captures initial attention significantly faster than AOI4, with an average difference of −503.100 ms, *p* < 0.001. Although AOI3’s TTFF is also lower than AOI4’s (difference of −3990.175 ms), this difference does not reach statistical significance (*p* = 0.071). Furthermore, the TTFF advantage of AOI1 relative to AOI3 is negligible and not statistically significant (Δ = −1062.925 ms, *p* = 0.876), indicating that proximity to the audience’s body (AOI1) immediately drives attention anchoring, rather than merely structural continuity. In the eye-tracking trajectory diagram, the first fixation location (Fixation Point 1) appears near AOI3, which is the point closest to the audience’s virtual position. This directly supports Hypothesis H3: In initial VR scene exploration, spatial proximity takes precedence over dynamic salience and distal continuity.

### 4.3. Correlate Subjective Experience with Grouping Effects

The eye-tracking metrics reveal patterns that indirectly reflect viewers’ subjective experiences of scene coherence and engagement. As Table 5 shows, spatial nodes (AOI1) and continuous structures (AOI3)—key drivers of successful spatial grouping (H1 & H2)—exhibited significantly higher cognitive processing depth, aligning with enhanced subjective coherence. Specifically, AOI1 showed markedly longer ADV compared to AOI4 (Δ = 2275.450 ms, d = 1.72), indicating prolonged engagement with this narrative endpoint. This suggests viewers perceived AOI1 as a coherent “meaning anchor,” fostering psychological closure. AOI3 elicited substantially higher TFD (Δ = 1962.500 ms, d = 3.65) and FC (Δ = 11.225, d = 3.88) relative to AOI4. The linear gaze trajectory along AOI3 (Figure 5) implies viewers used its continuity to construct narrative certainty, enhancing spatial coherence. Conversely, AOI4 demonstrated shorter visit durations (AOI3 vs. AOI4: Δ = −310.825 ms) and scattered fixations (Figure 5), indicating low engagement. This supports that dynamic elements unrelated to spatial goals failed to sustain attention or contribute to perceived coherence. These objective metrics—prolonged dwell times on structural nodes (AOI1) and focused scanning along paths (AOI3)—corroborate the implicit hypothesis: successful spatial grouping (closure, continuity) correlates with viewers’ subjective sense of narrative structure and immersion. Dynamic saliency (AOI4), lacking cognitive linkage to spatial intent, did not elicit comparable engagement. Ultimately, it was confirmed that spatial cognitive intentions, in VR environments, reshape the principles of perceptual organization, with dynamic saliency needing to align with user goals to effectively direct attention. Spatial cognitive intention refers to an internal activity of the mind or perceptual faculties aimed at representing and organizing spatial objects or spatial relationships. Its purpose is to enable the subject to understand space not merely as a physical extension, but as an entity that is structured and intelligible within consciousness. Overall, AOI1 exhibits a significantly greater visual appeal to viewers than all other AOI regions. AOI3 also performs robustly, slightly trailing AOI1 but notably outperforming AOI2 and AOI4. The visual appeal of AOI2 and AOI4 is insignificant, showing little distinction from other background areas.

## 5. Discussion

This study empirically demonstrates a fundamental shift in visual organization within VR films: spatial-intentional guidance overrides dynamic saliency in governing attention allocation. Eye-tracking results reveal that spatial nodes with clear functional significance and continuous structures dominate attention by activating adapted Gestalt principles such as closure, continuity, and proximity, whereas isolated dynamic elements fail to trigger automatic grouping. However, it is important to recognize that certain cognitive pathways and organizational mechanisms in VR films deviate from classical Gestalt laws. This observation aligns with emerging research in VR perception that highlights the importance of scene structure [69] but critically advances the field by establishing the primacy of goal-relevant spatial logic over bottom-up salience. Taken together, these findings underscore a shift toward cognitively driven visual organization in immersive environments. The following section synthesizes key insights, theoretical implications, and practical applications.

### 5.1. Space Node: As a Target-Oriented Predictive Cognitive Anchor Point

Spatial nodes in VR serve as active cognitive anchors, shaped by users’ bodily experience and goal anticipation. Users verify these nodes as narrative targets by turning their heads or shifting gaze, activating brain regions that integrate spatial and semantic information [70,71]. This aligns with predictive coding theories, where top-down expectations (e.g., identifying a dock as a goal) guide gaze to confirm predictions. The brain thus prioritizes goal-relevant validation over bottom-up saliency, explaining why isolated dynamic elements fail to attract attention unless they fit the spatial predictive model. Gaze duration on these nodes reflects the degree of goal certainty achieved [72]. Kim’s neuroimaging study [73] further confirms that identifying a spatial node activates the brain’s default mode network, linked to internal processing and goal confirmation. As a result, spatially meaningful nodes hold greater narrative power in VR than visually salient cues like color or brightness alone.

### 5.2. Continuity: Narrative Guidance Mechanism Based on Body Movements

In VR, continuity is shaped by the user’s embodied experience. When users follow a structure with their gaze, like tracing a bridge, they simulate bodily movement through space, activating neural pathways linked to visually guided motion [74,75]. Unlike contour recognition on 2D screens, VR continuity links the user’s immediate surroundings with distant narrative goals, turning Gestalt cues like continuity and closure into actionable guides. This body–space synchrony [76,77,78] makes continuity especially effective for maintaining attention. Conversely, dynamic elements not aligned with spatial intent (e.g., movement without directional meaning) are often ignored. This supports Slater’s view that VR realism arises from the integration of bodily movement with spatial meaning, not just visual motion [79].

### 5.3. Attention Shifting Mechanism: From Near-Object Anchoring to Target Control

In a VR environment, users initially attend to objects in close proximity, but this represents only the first phase in the process of shifting attention. The brain employs these nearby objects as anchors for self-localization [80], rapidly constructing an initial spatial representation of the environment. Immediately following this, attention transitions to a goal-directed control mode [81]. This shift involves activation of prefrontal brain regions responsible for planning and task prioritization [82]. Consequently, although users are initially drawn to nearby objects, their gaze ultimately shifts toward more distant, functionally relevant target nodes. This mechanism fundamentally operates through spatial cognitive intention—an internal process that organizes spatial relationships to construct coherent mental representations—which supersedes stimulus-driven saliency in governing attention allocation. This mechanism also clarifies why attention guidance strategies based on visual similarity (H4) or shared motion direction (H5) tend to fail in 360° panoramic VR contexts. First, the expansive field of view inherent to VR exceeds the brain’s capacity to rapidly integrate similar visual features [60], thereby compelling attention to rely predominantly on spatial continuity cues. Second, irrelevant motion signals are processed separately by the brain [83,84]; unless these movements carry cognitive significance, such as understanding that “water flows toward the pier,” which implies a navigable path, they are ineffective at guiding attention. Fundamentally, this attention transfer process operates as an efficient predictive system: proximal objects supply initial environmental cues (prior knowledge), while the user’s spatial goals (e.g., locating the pier) drive active exploration and hypothesis testing.

### 5.4. Practical Implications for VR Design

The empirical findings of this study advocate for a paradigm shift in VR content creation, urging designers to prioritize spatial intentionality over isolated visual salience when guiding user attention. Specifically, functional nodes should be strategically positioned as cognitive anchors, employing the Gestalt principle of closure to establish clear, explicit targets for user exploration and validation. Continuous spatial structures must be thoughtfully designed to adhere to Gestalt continuity principles, creating intuitive visual guidance corridors that resonate with users’ embodied sense of movement and structurally channel attentional flow from proximal to distal spatial domains. Dynamic elements in VR should only move when their motion aligns with spatial goals; otherwise, irrelevant movement may lead to unintended perceptual grouping or be ignored. Early-stage design should leverage proximity by placing simple, non-distracting elements near the viewer’s start point to support quick spatial orientation without competing with key spatial nodes or continuity paths. Effective VR design builds a coherent spatial narrative where every visual element, static or dynamic, supports goal-driven exploration, moving beyond 2D heuristics and bottom-up saliency to embrace the perceptual logic unique to immersive environments.

### 5.5. Research Limitations and Future Prospects

While this study provides substantial evidence supporting the applicability of Gestalt principles in VR films, several limitations must be acknowledged. First, the experimental stimulus consisted of a VR short film without a narrative plot, editing, or camera movement, featuring a fixed camera position. Although this design effectively controlled for confounding variables such as narrative complexity and sound, it somewhat limited the ecological validity of the findings. Second, the sample primarily comprised young college students, and the moderating effects of individual differences, such as spatial cognitive abilities, artistic proficiency, and cultural background, on Gestalt perception were not systematically explored. Although we considered participants’ frequency of VR experience, other relevant individual factors, including cognitive load tolerance and field dependence-independence, were not measured due to the study’s scope constraints. Furthermore, our VR experience categorization relied on self-reported frequency rather than objective metrics; future research should incorporate more rigorous and objective measures. The training protocol employed minimized novice effects but consequently precluded analysis of untrained, VR-naïve behavior, an intentional trade-off to ensure internal validity. Additionally, the sample predominantly consisted of young adults (50% aged 18–24), which may not fully represent perception patterns across a broader age demographic. As VR adoption continues to grow, future studies should expand participant age ranges to obtain more generalizable and accurate insights.

Given the study’s limitations, future research will focus on several areas: (1) improving the ecological validity of VR films by modeling perceptual interaction in real-world settings and testing Gestalt principles in more complex VR scenarios; (2) broadening the sample to include middle-aged and elderly participants and quantifying how individual differences, such as spatial cognition and cultural background, moderate perception, using physiological indicators to assess cognitive load and field dependence; (3) incorporating fNIRS/EEG neuroimaging to move beyond behavioral data and explore the neural basis of Gestalt grouping. Additionally, a VR narrative methodology grounded in spatial nodes and continuity will be developed to translate theoretical insights into creative practice.

## 6. Conclusions

This study uses eye-tracking to examine the applicability of classic Gestalt grouping principles in dynamic VR film scenes. Results reveal a shift in visual organization, driven more by users’ spatial cognitive intentions than by traditional visual saliency. Static elements with clear spatial functions effectively activate cognitive schemas and guide attention through Gestalt principles like closure, continuity, and proximity. In contrast, elements showing only visual similarity or motion coherence, without spatial relevance, fail to elicit strong grouping effects. Thus, the effectiveness of Gestalt principles in VR depends on the functional role of visual elements within the 3D narrative and their alignment with users’ cognitive goals. This highlights VR’s distinct perceptual logic, where visual storytelling relies on spatial coherence rather than on 2D or stimulus-based heuristics.

This study has certain limitations that should be acknowledged. Specifically, the assessment of participants’ cognitive function, motor abilities, and absence of mental illness and substance abuse history relied on observation and verbal questioning rather than standardized screening tools, which may introduce potential bias. Additionally, the consent process for the potential use of identifiable participant images involved obtaining retrospective consent, differing from ideal prospective consent practices for this specific purpose. However, despite these methodological limitations regarding screening and image consent procedures, this study provides robust empirical evidence for a fundamental shift in perceptual organization within VR, demonstrating for the first time that spatial cognitive intention supersedes dynamic saliency as the primary driver of visual attention allocation. These findings establish a novel paradigm for understanding and designing visual narratives in immersive environments.

## Figures and Tables

**Table 1:** The relationship between objective, hypotheses, Gestalt principle, and purpose.

Objective	Hypotheses	Gestalt Principle	Purpose
O1: Assess the applicability of Gestalt principles	H1	Closure	Validate if static spatial structures override dynamic saliency in guiding attention.
	H4	Similarity	Test if the similarity principle is ineffective in VR due to spatial discreteness.
	H5	Common Fate	Investigate whether dynamic grouping requires cognitive intent matching in VR.
O2: Examine VR’s impact on grouping efficiency	H2	Continuity	Quantify how spatial continuity guides gaze paths and suppresses irrelevant dynamics.
	H3	Proximity	Confirm the proximity law’s role in initial environmental orientation.
O3: Correlate subjective experience with grouping effects	Implicit in all hypotheses	N/A	Link eye-tracking metrics to psychological closure (H1) and narrative certainty (H2).

**Table 2:** Demographic characteristics of participants.

Category	Type	Sample Count	Percentage
Gender	Male	25	59.5%
	Female	17	40.5%
Age Range	18~24 years old	21	50.0%
	24~30 years old	15	35.7%
	30~36 years old	6	14.3%
Familiarity with VR Technology	Never did	3	7.1%
	A little	32	76.2%
	Very well	7	16.7%
Familiarity with VR Films	Never did	13	31.0%
	Occasionally	26	61.9%
	Often	3	7.1%

**Table 3:** Multiple comparison results of eye movement indicators (TFD, FC, SAC) for evaluating Gestalt principles applicability.

Eye-Tracking Indicators	Group Comparison of AOI	Mean Difference	Std. Error	Significance	Cohen’s d	95% CI	
						Lower	Upper
TFD	AOI1 vs. AOI2	5085.200 *	1044.999	<0.001	3.12	2316.89	7853.51
	AOI1 vs. AOI4	5176.525 *	985.241	<0.001	3.45	2548.95	7804.1
	AOI2 vs. AOI4	91.325	517.865	0.998	0.12	−1274.3	1456.95
FC	AOI1 vs. AOI4	16.675 *	3.178	<0.001	2.78	8.27	25.08
	AOI2 vs. AOI4	−1.625	2.568	0.921	−0.34	−8.39	5.14
SAC	AOI1 vs. AOI4	9.850 *	2.141	<0.001	2.45	4.15	15.55
	AOI4 vs. AOI3	−3.350 *	0.705	<0.001	−2.52	−5.22	−1.48

Note: * The significance level of the average difference is 0.05. These data were analyzed using the Geems–Howell method.

**Table 4:** Multiple comparison results of eye movement indicators (SAC and TIFF) to examine VR’s impact on grouping efficiency.

Eye-Tracking Indicators	Group Comparison of AOI	Mean Difference	Std Error	Significance	Cohen’s d	95% CI	
						Lower	Upper
SAC	AOI3 vs. AOI4	3.350 *	0.705	<0.001	2.52	1.48	5.22
TTFF	AOI1 vs. AOI4	−5053.100 *	1143.031	<0.001	−3.45	−8066.4	−2039.8
	AOI3 vs. AOI4	−3990.175	1604.706	0.071	−1.73	8209.74	229.39
	AOI1 vs. AOI3	−1062.925	1415.748	0.876	−0.51	−4812.3	2686.45

Note: * The significance level of the average difference is 0.05. These data were analyzed using the Geems–Howell method.

**Table 5:** Multiple comparison results of eye movement indicators (TFD, FC, and ADV) correlate subjective experience with grouping effects.

Eye-Tracking Indicators	Group Comparison of AOI	Mean Difference	Std. Error	Significance	Cohen’s d	95% CI	
						Lower	Upper
TFD	AOI3 vs. AOI4	1962.500 *	288.938	<0.001	3.65	1194.35	2730.65
FC	AOI3 vs. AOI4	11.225 *	1.545	<0.001	3.88	7.13	15.32
SAC	AOI1 vs. AOI4	2275.450 *	720.501	<0.001	1.72	346.588	4204.312
	AOI3 vs. AOI4	−310.825 *	185.236	<0.001	−0.92	−797.444	175.794

Note: * The significance level of the average difference is 0.05. These data were analyzed using the Geems–Howell method.

## Data Availability

The original contributions presented in the study are included in the article; further inquiries can be directed to the corresponding author.
